# Combining Transoral Incisionless Fundoplication and Endoscopic Sleeve Gastroplasty (F-ESG): An Endoscopic Approach to Treat Pathologic Gastroesophageal Reflux in Obesity

**DOI:** 10.1007/s11695-026-08526-3

**Published:** 2026-02-18

**Authors:** Maryam Alkhatry, Jamil Samaan, Barham Abu Dayyeh

**Affiliations:** 1 Department of Gastroenterology, Obaidullah Hospital, Emirates Health Services, Ministry of Health, Ras Al Khaimah, United Arab Emirates; 2 Karsh Division of Gastroenterology and Hepatology, Cedars-Sinai Medical Center, Los Angeles, United States

**Keywords:** Gastroesophageal reflux disease, Obesity, Endoscopic sleeve gastroplasty, Transoral incisionless fundoplication, Bariatric endoscopy

## Abstract

**Background and Aims:**

Obesity-related Gastroesophageal Reflux Disease (GERD) presents a significant clinical challenge, limited by the effectiveness of current treatments. Excess weight impairs the repair of the anti-reflux barrier, and conventional obesity treatments may adversely affect anti-reflux physiology. This study systematically evaluates a modular endoscopic technique that combines Transoral Incisionless Fundoplication (TIF) with Endoscopic Sleeve Gastroplasty (ESG) simultaneously (F-ESG), offering a synergistic solution to both GERD and obesity.

**Methods:**

This single-center pilot study enrolled adults with objectively confirmed pathologic GERD and incomplete symptomatic response to proton pump inhibitor therapy. Participants underwent the combined F-ESG procedure with standardized dietary and behavioral counseling. Outcomes were assessed at baseline, 6 months, and 12 months, including percent total weight loss (%TWL), GERD Health-Related Quality of Life (HRQL), Reflux Symptom Index (RSI), and DeMeester score (DMS) obtained from 48-hour pH monitoring.

**Results:**

Eight participants (mean age 39 ± 6.4 years; 75% female; mean BMI 34.5 ± 3.7 kg/m²) were included. Mean %TWL was 13.1% at 6 months and 15.2% at 12 months. GERD HRQL improved from 26.4 at baseline to 8.3 at 6 months and 8.5 at 12 months (*p* < 0.01). RSI and DMS also demonstrated significant reductions. By 12 months, all participants had discontinued PPI therapy, and 7 of 8 achieved a normalized DeMeester score.

**Conclusions:**

In this single-center pilot study, same-session F-ESG was feasible and safe, with improvements in subjective and objective GERD metrics and weight loss through 12 months. Larger multicenter comparative studies are warranted to evaluate efficacy and durability.

**Supplementary Information:**

The online version contains supplementary material available at 10.1007/s11695-026-08526-3.

## Key Points

• F-ESG was feasible and safe in patients with obesity and medically refractory GERD.

• F-ESG improved subjective and objective GERD and achieved meaningful weight loss.

• These pilot findings support further multicenter studies of durability and efficacy.

## Introduction

Gastroesophageal reflux disease (GERD) and obesity represent two significant and interrelated public health challenges with increasing prevalence worldwide [1]. The pathophysiological relationship between these conditions is well-established, with obesity being a major risk factor for GERD development and exacerbation [2, 3]. Excess adipose tissue increases intra-abdominal pressure, promoting gastroesophageal reflux through mechanical and pathophysiologic disruption of the anti-reflux barrier [4]. 

Current treatment approaches for obesity-related GERD present significant limitations. Pharmacological management with proton pump inhibitors (PPIs) provide incomplete symptom relief and does not address the underlying anatomical and pathophysiological abnormalities [5, 6]. Conventional bariatric surgery procedures, while effective for weight loss, may have variable effects on GERD symptoms. Notably, sleeve gastrectomy has been associated with new-onset or worsening GERD in a substantial proportion of patients [7, 8]. Comparative studies have demonstrated GERD rates of 1.9% in Roux-en-Y gastric bypass versus 14.5% in sleeve gastrectomy patients (*P* < 0.05) [9]. Similarly, meta-analyses have reported GERD prevalence of 1.3% in bypass procedures compared to 17.9% in sleeve gastrectomy (*P* < 0.05) [10].

The complex interplay between obesity and GERD necessitates innovative approaches that simultaneously address both conditions while minimizing adverse effects. Endoscopic bariatric and metabolic therapies have emerged as minimally invasive alternatives to traditional surgical interventions, offering reduced procedural risks, organ preservation, and improved recovery time [11]. Among these, Endoscopic Sleeve Gastroplasty (ESG) has demonstrated efficacy for weight loss, while Transoral Incisionless Fundoplication (TIF) has shown efficacy for GERD management [12, 13]. 

This study evaluates a combined approach, Fundoplication-ESG (F-ESG), that integrates TIF and ESG in a single endoscopic session. The F-ESG technique aims to augment the anti-reflux barrier through fundoplication while simultaneously restricting gastric volume through gastroplasty, preserving anatomy and minimizing disruption to normal physiology. This modular endoscopic approach represents a potential paradigm shift in managing the obesity-GERD complex, offering a synergistic solution to both conditions without the limitations of current treatments.

## Methods

### Study Design

This prospective pilot study was designed to assess the feasibility, safety, and 12-month reflux and weight outcomes of same-session combined TIF and ESG (F-ESG). The study was conducted between January 2022 and February 2023. All participants provided written informed consent and were entered into a dedicated registry. The study adhered to the Declaration of Helsinki and was approved by the institutional ethics committee.

### Participants

Inclusion criteria comprised: (1) age 18–65 years; (2) body mass index (BMI) 30–40 kg/m²; (3) documented pathologic acid reflux on 48-hour pH monitoring (DeMeester score > 14.7); (4) GERD symptoms despite PPI therapy; and (5) absence of large (> 2 cm) hiatal hernia on endoscopy. Exclusion criteria included: (1) previous gastric or esophageal surgery; (2) Barrett’s esophagus; (3) esophageal motility disorders; (4) pregnancy; (5) contraindications to endoscopy; (6) LA Grade C-D esophagitis, and (7) inflammatory, neoplastic, or vascular esophagogastric lesions. All patients underwent pre-procedural barium esophagram without evidence of abnormal esophageal motility, and none reported symptoms suggestive of a motility disorder.

### Procedure

All procedures were performed under general anesthesia by two endoscopists experienced in both TIF and ESG techniques. The F-ESG procedure was conducted in two sequential steps during the same endoscopic session:

#### Transoral Incisionless Fundoplication (TIF)

Using the EsophyX Z device (EndoGastric Solutions, Redmond, WA), the gastroesophageal junction was reconstructed by creating a 270° partial fundoplication with approximately 20 fasteners placed above the Z-line. This step aimed to restore the angle of His and augment the anti-reflux barrier.

#### Endoscopic Sleeve Gastroplasty (ESG)

Following TIF completion, ESG was performed using the OverStitch endoscopic suturing system (Apollo Endosurgery, Austin, TX) using a double channel-endoscope. The greater curvature of the stomach was plicated with approximately 6–8 full-thickness sutures in a U-shaped suturing pattern from the distal-to-proximal stomach, creating a sleeve-like restriction of the gastric lumen [14]. The fundal reservoir was reduced given that the TIF valve occupied a portion of the fundus; however, no additional technical modifications were required. No issues related to scope maneuverability or device passage were encountered, and the presence of the fundoplication did not alter the suturing pattern or proximal plication strategy.

The sequence of procedures was chosen to optimize the technical integrity of both components. TIF was performed first to maintain the native fundic anatomy needed for creation of an effective gastroesophageal valve. Completing TIF prior to gastric remodeling avoids the restriction of fundic mobility and the increased axial tension that occur after ESG, both of which can impair valve formation. Furthermore, performing ESG first would reduce intragastric space, which can impair visibility and limit maneuverability of the EsophyX Z device. A representative case video of the F-ESG procedure, demonstrating the sequential performance of TIF followed by ESG in a single session, is provided as supplementary material (Supplementary Video 1).

### Post-Procedure Management and Follow-up

All patients were observed overnight and discharged the following day if clinically stable. Patients followed a standard post-bariatric diet progression: clear liquids for 3 days, full liquids for 2 weeks, pureed foods for 2 weeks, and then gradual transition to solid foods. All participants received nutritional counseling and were advised to follow a low-calorie diet (approximately 1200 kcal/day) with moderate physical activity (150 min/week). PPI therapy was continued for 6 weeks post-procedure and then tapered off. Follow-up evaluations occurred at baseline, 6 months, and 12 months post-procedure and included:

- Anthropometric measurements: weight, BMI, and percent total weight loss (%TWL).
- GERD symptom evaluation: GERD Health-Related Quality of Life (HRQL) questionnaire and Reflux Symptom Index (RSI).
- Objective GERD assessment: 48-hour pH study off proton pump inhibitor therapy and DeMeester score.
- Medication use: assessment of PPI requirement and dosage.

### Statistical Analysis

Descriptive analyses were performed, with categorical variables reported as counts and percentages, while continuous variables were reported as means or percentages ± standard deviations, as appropriate. Procedure time, technical success, and immediate adverse events were recorded. Changes from baseline were analyzed using paired t-tests for continuous variables and McNemar’s test for categorical variables. Given the small sample size, Wilcoxon signed-rank tests were additionally performed as sensitivity analyses for paired comparisons. Results were considered exploratory. Statistical significance was set at *P* < 0.05. All analyses were performed using SPSS version 26.0 (IBM Corp., Armonk, NY).

## Results

The study cohort comprised 8 patients (6 females, 2 males) with a mean age of 39 ± 6.4 years and mean BMI of 34.5 ± 3.7 kg/m² (Table 1). All patients had pathologic GERD with a mean DeMeester score of 50 ± 39.4, mean GERD HRQL score of 26.4 ± 9.6, and mean RSI of 21 ± 8.9 at baseline. All participants were on daily PPI therapy before the procedure. Nonparametric sensitivity analyses using Wilcoxon signed-rank testing yielded results consistent with the primary paired analyses.

**Table 1:** Baseline characteristics and 12-month outcomes after combined transoral incisionless fundoplication and endoscopic sleeve gastroplasty (F-ESG)

*N*	Baseline	12 Months	*P*-value
	8	8	
Female Sex, n (%)	6 (75%)	—	
Age (Years)	39 ± 6.4		
BMI (Kg/m^2)	34.5 ± 3.7		
Daily PPI Therapy	8 (100%)	0	
% Total Weight Loss (%TWL)	—	15.2 ± 3.8	*P* < 0.01
DeMeester Score	50.0 ± 39.4	6.6 ± 4.4	*P* < 0.01
GERD-HRQL Score	26.4 ± 9.6	8.5 ± 6.6	*P* < 0.01
Reflux Symptom Index (RSI)	21.0 ± 8.9	9.3 ± 8.7	*P* < 0.01

*SD* Standard deviation; *BMI* Body mass index; *PPI* Proton Pump Inhibitor. *GERD-HRQL* Gastroesophageal reflux disease health-related quality of life

### Procedural Outcomes

Technical success was achieved in all 8 patients, with successful completion of both TIF and ESG components. The mean procedure time was 95 ± 15 min. No immediate major adverse events were observed. Minor adverse events included post-procedure pain (*n* = 3) and nausea (*n* = 2), which resolved with conservative management.

### Weight Loss Outcomes

**Fig. 1:**
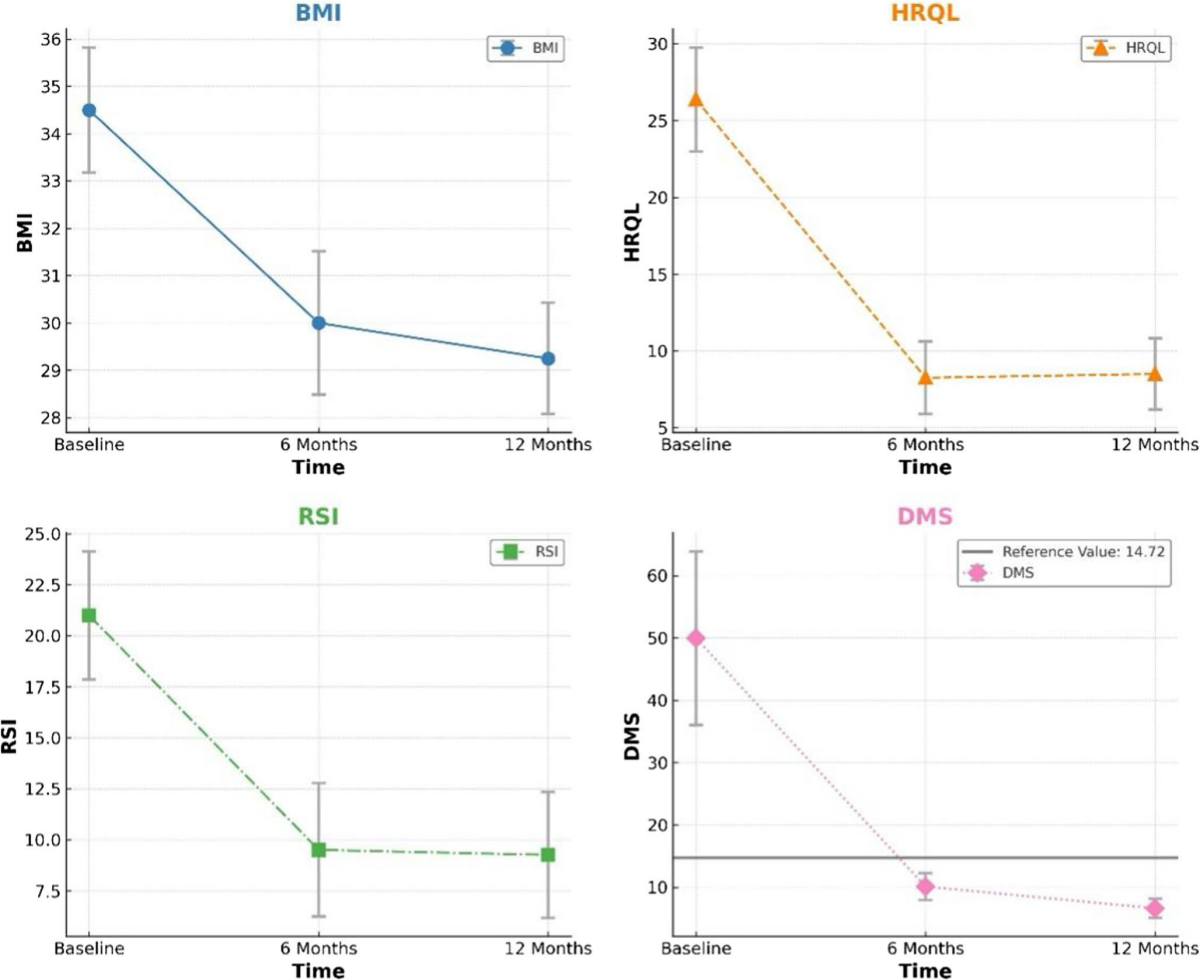
Change in Body Mass Index (BMI), Health-Related Quality of Life (HRQL), Reflux Symptom Index (RSI), and DeMeester Score (DMS) at Baseline, 6, and 12 Months After Combined Transoral Incisionless Fundoplication with Endoscopic Sleeve Gastroplasty (F-ESG)

Significant weight loss was observed throughout the follow-up period. Mean BMI decreased from 34.5 ± 3.7 kg/m² at baseline to 30.0 ± 3.2 kg/m² at 6 months and 29.3 ± 3.0 kg/m² at 12 months (*P* < 0.01 for both comparisons). The mean %TWL was 13.1 ± 3.2% at 6 months and 15.2 ± 3.8% at 12 months (Fig. 1, Supplementary Fig. 1)

### Subjective GERD Measures

GERD symptoms showed substantial improvement following the F-ESG procedure. The mean GERD HRQL score decreased from 26.4 ± 9.6 at baseline to 8.3 ± 6.7 at 6 months and 8.5 ± 6.6 at 12 months (*P* < 0.01 for both comparisons) (Fig. 1). Similarly, the mean RSI improved from 21 ± 8.9 at baseline to 9.5 ± 9.3 at 6 months and 9.3 ± 8.7 at 12 months (*P* < 0.01 for both comparisons) (Fig. 1). Individual patient outcomes are shown in Supplementary Table 1.

### Objective GERD Measures

Objective improvement in GERD was demonstrated by significant reductions in acid exposure. The mean DeMeester score decreased from 50 ± 39.4 at baseline to 10.1 ± 6.1 at 6 months and 6.6 ± 4.4 at 12 months (*P* < 0.01 for both comparisons) (Fig. 1). By 12 months, 7 of 8 patients (87.5%) had achieved normalization of their DeMeester score (< 14.7). Individual patient outcomes are shown in Supplementary Table 1.

### Medication use

All patients were able to discontinue PPI therapy by 6 months post-procedure and remained off PPIs at the 12-month follow-up. One patient occasionally used over-the-counter antacids for breakthrough symptoms. Individual patient outcomes are shown in Supplementary Table 1.

### Adverse Events

No serious adverse events were reported during the 12-month follow-up period. Two patients experienced dysphagia in the first month post-procedure, which resolved spontaneously without intervention. No patients required reoperation or endoscopic revision.

## Discussion

This pilot study demonstrates that the F-ESG approach, combining TIF with ESG in a single endoscopic session, is technically feasible and potentially effective for the management of obesity-related GERD. The procedure resulted in significant improvements in both objective and subjective measures of GERD, along with clinically meaningful weight loss, suggesting a synergistic effect in addressing these interrelated conditions.

The pathophysiological relationship between obesity and GERD is well-established, with multiple mechanisms contributing to reflux in individuals with obesity [15]. Weight loss is known to improve GERD symptoms, but conventional bariatric procedures have variable effects on reflux. Sleeve gastrectomy, despite its popularity and efficacy for weight loss, has been associated with new-onset or worsening GERD in up to 20% of patients [16]. This is attributed to alterations in gastric anatomy, including increased intragastric pressure and disruption of the angle of His [17]. While Roux-en-Y gastric bypass is considered more favorable for GERD management, it involves significant anatomical rearrangement and potential long-term nutritional concerns [18]. 

The F-ESG approach represents a new paradigm that addresses both obesity and GERD through complementary endoscopic techniques while preserving normal anatomy. TIF restores the angle of His and augments the anti-reflux barrier, while ESG restricts gastric volume and promotes weight loss without altering the fundamental gastric anatomy. This modular approach allows for tailored treatment based on the predominant pathology while minimizing the risk of worsening either condition.

While ESG is associated with a significantly lower incidence of postoperative GERD compared with sleeve gastrectomy, published series still report de novo reflux in a minority of patients, with rates ranging from 0–4.3% [19–23]. Notably, these studies include general ESG populations who were not selected for severe or PPI-refractory reflux. In contrast, the patients in our study all demonstrated pathologic acid exposure and persistent symptoms despite optimized medical therapy prior to F-ESG. In this high-risk patient population, the reduction of intragastric pressure from the weight loss alone may be insufficient to achieve durable reflux control given the etiology of GERD is multifactorial and may reflect both the impact of obesity as well as other anatomical contributors to barrier incompetence. Although the independent postprocedural effect of ESG-associated weight loss on reflux outcomes has not been clearly defined, data from non-surgical weight-loss interventions provide insight into the limits of weight reduction alone. Prior data suggest that weight loss alone may not reliably normalize reflux in medically refractory GERD [24]. The combined ESG–TIF approach directly addresses this therapeutic gap by pairing gastric remodeling with immediate augmentation of the antireflux barrier in a single session, thereby reducing the need for staged interventions.

Shah et al. previously reported the first case of same-session ESG and TIF [25]. Our study expands upon this initial experience by presenting the first case series with systematic follow-up and objective reflux measurements. Our findings are consistent with previous studies of TIF alone, which have demonstrated efficacy in reducing acid exposure and improving quality of life [26]. However, the addition of ESG appears to enhance these outcomes through weight loss, potentially addressing a key pathophysiological factor in obesity-related GERD. The observed weight loss in our cohort is comparable to that reported in studies of ESG alone [27], suggesting that the combined procedure does not compromise the weight loss efficacy of ESG.

The ability of all patients to discontinue PPI therapy by 6 months post-procedure is particularly noteworthy, as it addresses a significant limitation of pharmacological management. Long-term PPI therapy has been associated with adverse outcomes in observational studies [28]. The F-ESG approach offers a potential alternative that may reduce or eliminate the need for lifelong medication.

The safety profile observed in our study is encouraging, with no serious adverse events and only minor, self-limiting complications. This is consistent with the established safety profiles of both TIF and ESG when performed individually [29, 30]. The combined procedure did not appear to increase the risk of adverse events, suggesting that the integration of these techniques is feasible from a safety perspective.

Several limitations of this study should be acknowledged. The sample size was small (*n* = 8) and derived from a single center, limiting external validity and generalizability. The absence of a comparator group limits comparison with alternative treatment approaches as well as precludes conclusions regarding the independent contribution of ESG versus TIF to the observed outcomes. The lack of endoscopic follow-up data prevents anatomical assessment of TIF and ESG constructs and therefore prevents correlation with physiologic response and limits mechanistic assessments to directly evaluate changes in gastroesophageal junction physiology or valve integrity, limiting mechanistic inference. Although 12-month follow-up is appropriate for an initial feasibility study, longer-term follow-up is needed to assess durability of reflux control and weight loss. Accordingly, our findings should be interpreted as hypothesis-generating and require confirmation in larger, multicenter comparative studies. To ensure safe and effective implementation into clinical practice, future larger studies should formally evaluate learning curves, inter-operator variability, suture patterns and reproducibility across centers.

## Conclusions

In this single-center pilot study, combined transoral incisionless fundoplication and endoscopic sleeve gastroplasty (F-ESG) was feasible and safe and was associated with improvement in objective and symptom-based gastroesophageal reflux outcomes and clinically meaningful weight loss through 12 months. These findings offer a promising, minimally invasive option for addressing the complex relationship between obesity and GERD. Larger, multicenter comparative studies are required to confirm efficacy and evaluate durability.

## Supplementary Information

Below is the link to the electronic supplementary material.

ESM 1 Supplementary Video 1: Endoscopic Sleeve Gastroplasty With Transoral Fundoplication (F-ESG): Technique and Case Demonstration (MOV 358 MB)

ESM 2 (DOCX 149 KB)

## Data Availability

The data underlying this study are not publicly available due to institutional policies and patient confidentiality. Deidentified data may be made available from the corresponding author upon reasonable request.

## Abbreviations

BMI: Body mass index
ESG: Endoscopic sleeve gastroplasty
F-ESG: Fundoplication-Endoscopic Sleeve Gastroplasty
GERD: Gastroesophageal Reflux Disease
HRQL: Health-Related Quality of Life
PPIs: Proton Pump Inhibitors
RSI: Reflux Symptom Index
TIF: Transoral Incisionless Fundoplication
