# Supplementary material for: Combining Transoral Incisionless Fundoplication and Endoscopic Sleeve Gastroplasty (F-ESG): An Endoscopic Approach to Treat Pathologic Gastroesophageal Reflux in Obesity
Source: Obes Surg. 2026 Feb 18;36(3):1264–70. doi: 10.1007/s11695-026-08526-3 (PMC13038680; doi:10.1007/s11695-026-08526-3)
Supplement: Supplementary file 2 — (DOCX 149 KB) [file 11695_2026_8526_MOESM2_ESM.docx]

**Supplementary Table 1:** Patient-level Reflux and Weight Outcomes Through 12 Months After F-ESG.

|  | **%TWL** | **DeMeester Score** | | **HRQL** | | **RSI** | | **PPI Use** | |
| --- | --- | --- | --- | --- | --- | --- | --- | --- | --- |
| **Subject** | **12 Months** | **Baseline** | **12 Months** | **Baseline** | **12 Months** | **Baseline** | **12 Months** | **Baseline** | **12 Months** |
| 1 | 13.51 | 9.4 | 2.2 | 24 | 14 | 26 | 11 | Yes | No |
| 2 | 15.79 | 22.2 | 5.1 | 27 | 7 | 24 | 9 | Yes | No |
| 3 | 25.0 | 31.4 | 6.4 | 26 | 16 | 24 | 18 | Yes | No |
| 4 | 25.81 | 103.3 | 2.2 | 15 | 2 | 12 | 1 | Yes | No |
| 5 | 13.33 | 31.4 | 15.4 | 41 | 5 | 33 | 8 | Yes | No |
| 6 | 15.15 | 118.7 | 6 | 15 | 5 | 6 | 2 | Yes | No |
| 7 | 0 | 36.2 | 10.5 | 39 | 1 | 27 | 0 | Yes | No |
| 8 | 11.11 | 47 | 5.2 | 24 | 18 | 16 | 25 | Yes | No |

**TWL:** Total Weight Loss; **HRQL:** Health-Related Quality of Life; **RSI:** Reflux Symptom Index; **PPI:** Proton Pump Inhibitor.

***
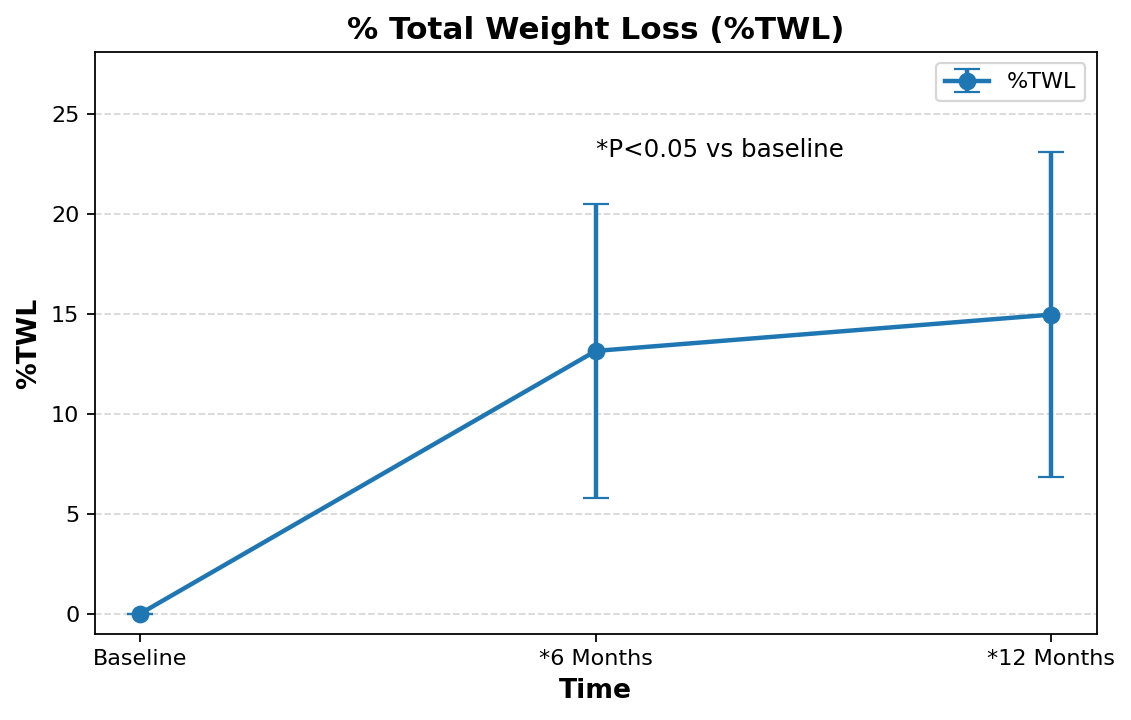
***

**Supplementary Figure 1:** Change in Percent Total Weight Loss (%TWL) at Baseline, 6, and 12 Months After Combined Transoral Incisionless Fundoplication with Endoscopic Sleeve Gastroplasty (F-ESG).
